# Effectiveness of Pilates Training on Body Composition and Isokinetic Muscular Strength in Adolescent Baseball Players

**DOI:** 10.3390/ijerph191912085

**Published:** 2022-09-24

**Authors:** Jang Soo Yook, Da Yoon Kim, Dong Hun Choi, Min-Seong Ha, Yoon Young Hwang

**Affiliations:** 1 Brain Research Institute, Korea Institute of Science and Technology, 5 Hwarang-ro 14-gil, Seongbuk-gu, Seoul 02792, Korea; 2 Faculty of Health and Sport Sciences, University of Tsukuba, 1-1-1 Tennodai, Tsukuba 305-8574, Ibaraki, Japan; 3 Department of Sport Medicine, Konyang University, 121 Daehak-ro, Chungcheongnam-do, Nonsan-si 32992, Korea; 4 Department of Sports Culture, College of the Arts, Dongguk University-Seoul, 30 Pildong-ro 1-gil, Jung-gu, Seoul 04620, Korea; 5 Exercise Biochemistry Laboratory, Korea National Sport University, 1239 Yangjae-daero, Songpa-gu, Seoul 05541, Korea

**Keywords:** Pilates training, adolescent baseball players, body composition, isokinetic muscular strength

## Abstract

Body composition and muscular strength are important for baseball skills and successful performance. Conditioning training programs have the potential to enhance athletic performance via physiological changes. In this single-group interventional study, we investigated the effect of 8 weeks of Pilates training (PT) on contralateral and regional body composition, and isokinetic muscular strength in knee and trunk flexion/extension in adolescent baseball players. In our results, PT increased both right- and left-sided lean mass in the trunk. Following PT, work per repetition and average power showed significant increases in the flexion and extension of the left knee only. PT significantly decreased the peak torque of the trunk flexor and increased the average power of the trunk extensor. In addition, the ratio of the trunk flexion/extension strength of peak torque showed a decreasing trend, whereas that of work and average power did not change significantly following PT. In conclusion, PT evenly improved lean mass on both the right and left sides of the body. Knee and trunk strength increased after PT. Our findings suggest that PT may be a useful strategy for enhancing athletic performance in regard to the muscular strength of adolescent baseball players.

## 1. Introduction

Adolescents worldwide are involved in various sports and physical activities. Parallel to the growing participation in sports, a long-term athlete development model has been recommended to ensure a suitable level of growth, maturation, and training in adolescence. This model may have an important role in the improvement of fundamental motor skills, contributing toward success in competitive adult sports [1,2]. Fitness coaches have used various types of exercise, including endurance, strength, and flexibility training, to reach adequate physical maturation and development [3,4]. Such conditioning exercise programs, with proper supervision and plans, should be implemented to improve sports-related physiological factors, such as body composition and muscular strength.

Body composition refers to the absolute and relative amounts of bone, muscle, and fat mass as a characterization of body weight, which is an important determinant of athletic ability and performance [5]. Body composition can change during the baseball season [6], and these alterations may influence performance and injury presentation. Its measurement or evaluation also plays an essential role in monitoring the potential effects of training programs on adolescent baseball players [7]. Lean mass (LM) is an essential body component that plays an important role in maintaining normal movement and physical performance in adolescents [8]. In other sports, changes in LM have been associated with muscle power and strength in adolescent athletes [9,10]. Similarly, a recent study demonstrated that higher LM is positively associated with greater muscle strength and power [11]. Therefore, the optimization of body composition, in particular of LM, in response to such conditioning training programs may be an important factor for adolescent players to enhance their athletic performances without injury.

Sports such as baseball require high levels of muscular strength to maximize athletic performance. Higher muscular strength and power are also associated with a lower risk of injury in both training and competition [12]. In baseball players, throwing motions require total body kinematics with fine coordination of body segments involving the upper and lower limbs and the trunk [13,14]. As maximal throwing speed is required, specific muscle strength balance contributes to optimal performance, as well as prevention of overuse injuries in adolescent players [15,16]. In particular, the trunk muscles play an important role in the transfer of momentum and torque from the lower extremities during pitching, which involves acceleration of the arm [17,18]. However, improper trunk motion with excessive contralateral trunk leaning has been associated with a potential risk for upper limb injuries in adolescent players [17,19]. Given the importance of trunk stability in baseball players, improved muscle strength, particularly in the trunk and lower limbs, may contribute to improved athletic performance.

Several types of training have been used to improve physical conditions in various sports. Among these, Pilates training (PT) as a novel type of exercise program has provided beneficial effects in psychological functions, achieving mind concentration and physiological functional performance, and increasing core muscle stability [20,21]. In fact, Pilates exercise is a whole-body conditioning program based on key components, such as concentration, control, precision, flow, breathing, and centering [22], which have been widely used in health and fitness [23]. PT is composed of muscular stretching and strengthening exercises to improve strength, endurance, coordination, balance, and flexibility, which may be useful for enhancing athletic performance [24]. Several studies have reported the positive effects of PT on muscular strength. In particular, PT seems to be effective in improving the trunk muscles. Endleman et al. reported that PT increases stability and activates the deep muscles of the trunk [25]. Gonzalez-Calvez et al. demonstrated that a 6-week program of PT could improve trunk strength in adolescents with a history of back pain [26].

Core stability is an important issue in athletes. During pitching and throwing movements in baseball, the upper extremity movement comes through the trunk and pelvic girdle; therefore, appropriate trunk mobility is important for good performance [27]. Weakened core stability can cause lower back pain, which can lead to a decrease in flexibility, range of joint motion, muscle endurance, and strength due to reduced physical activity caused by pain [28]. Since activation of trunk stabilizers can promote more efficient sports activities [14], the beneficial effects of PT could also contribute to improving the stability and performance of athletes [29].

In addition to its effect on muscular strength, PT also induces a beneficial change in body composition. Recent systematic reviews reported that PT improved body weight, fat mass, and lean mass, as well as outcome parameters of obesity [30,31]. Most studies focused on the positive effects of PT in general healthy individuals. Although a recent previous study suggested that PT may have a potential role in improving body composition in adolescent athletes [32], further evidence-based studies are required to corroborate this hypothesis.

Given the reported advantage of PT, this study aimed to investigate the effects of supervised PT on changing body composition and muscular strength of the trunk and knee in adolescent baseball players. We hypothesized that an 8-week PT program would enhance body composition and isokinetic muscular strength in the lower limbs.

## 2. Materials and Methods

### 2.1. Procedures

The experimental design and protocol of this study were approved by the Institutional Review Board (IRB) Committee of Korea National Sport University (IRB No. 20200212-107) before the beginning of the research project. This study, for research into sports medicine, was performed according to the guidelines of the Declaration of Helsinki. All adolescent players and their legal guardians (such as parents) were given information about the study and provided informed consent before participation in the study training program. All participants were informed that they could withdraw from the experiment at any time. Following the training program, the players underwent 8 weeks of PT (Table 1), which took place at the same time of the day (4:00–5:00 p.m.). All measurements were performed once at the Korea National Sports University laboratory before and after the 8-week block of PT. All the participants were instructed not to ingest food for two hours before the test. All assessments were performed in the same order (body composition–knee strength–trunk strength) on both days.

### 2.2. Participants

Male middle school baseball players older than 15 years participated in this study. Taking into consideration an error of 20%, an a value of 0.05, and effect size (*d* = 1.0) with G*Power 3.1 software (Kiel University, Kiel, Germany), 11 participants were required for this study. All participants competed in the Korea Baseball Softball Association league and had at least 5 years of baseball experience within the youth academy of a professional baseball team. Participants with neuromuscular disease were excluded from the study. Most participants were right-handed throwing players, with one left-handed throwing player, and all participants were right-limb dominant.

### 2.3. Pilates Training Program Intervention

All participants in this study were trained using the PT program described in a previous study [32]. The 8 weeks of PT was performed during off-season baseball training to minimize the effect of regular baseball training and competition. The participants completed 8 weeks of training with thrice-weekly sessions. The PT training consisted of a 5 min warm up, a 40 min main workout, and a 5 min cool-down exercise, and it took place over 3 days (Monday, Wednesday, and Friday) as shown in Table 1. Incremental Pilates motion was applied with a progressive increase in exercise intensity. During the intervention period, Pilates experts and an exercise physiologist instructed and supervised the participants.

### 2.4. Evaluation

Body composition before and after PT was evaluated by measuring participant height (cm) and weight (kg) in light clothes (Dong-Sahn Jenix, Seoul, Korea). An X-ray meter (dual-energy X-ray absorptiometry, DEXA, GX system, Madison, WI, USA) was used to assess the components of body composition, including the percentage of fat (%), fat mass (kg), lean mass (kg), fat-free mass (kg), bone mineral content (BMC, kg), and bone mineral density (BMD, g/cm^2^).

Isokinetic muscle strength tests before and after PT were conducted using Humac NORM isokinetic dynamometers (CSMi; Stoughton, MA, USA). Participants performed the concentric strength of knee extensors/flexors test and then the trunk extensors/flexor strength test. A range of motion (ROM) for flexion and extension of the knee and trunk was set from 0° of extension to 90° of flexion. To avoid the influence of fatigue, participants rested for 30 min between tests.

#### 2.4.1. Body Composition

An experienced technician performed all DEXA assessments. Before measurement, participants were asked to remove all metal, heavy plastic items and thick clothing to avoid interference with the DEXA scan. All participants were instructed to lie in the supine position on the scanning table, and continuous transverse imaging was subsequently conducted for 10 min.

#### 2.4.2. Knee Flexion/Extension

Participants were seated on an isokinetic dynamometer with 90° flexion of the hip and the knee joints. The participant’s trunk was fixed to the backrest of the dynamometer to avoid compensatory upper-body movements. The dynamometer axis of rotation was aligned with the rotational axis of the knee. A lever arm pad was placed slightly proximal to the medial malleolus, 0° was determined as 0° knee extension, and tests were per-formed with a ROM of 90° to 0°. The knee extension and flexion of the peak torque, work per repetition, and average power per repetition in each leg were concentrically conducted five times at 60°/s [33].

#### 2.4.3. Trunk Flexion/Extension

Participants stood on the footplate of the dynamometer, and the scapular and chest pads were fastened in parallel across the center of the scapulae and against the subject’s chest. The feet were placed in a fixed position against the footplate heel cups, separated by shoulder width. The thigh pad, tibial pad, and pelvic belts were attached to help secure the lower body firmly, minimizing the confounding effect of other muscles during trunk extension. The dynamometer axis of rotation was aligned with a line on the iliac crest. Each repetition started from the flexion position. A ROM of flexion and extension of the trunk was set from 0° of extension to 90° of flexion. The trunk extension and flexion of the peak torque, work per repetition, and average power per repetition were concentrically conducted three times at 30°/s [34].

### 2.5. Statistical Analysis

The mean values and standard deviations were used for statistical analysis. The Shapiro–Wilk test indicated that all variables were normally distributed. A dependent *t*-test was performed to investigate the effects of 8 weeks of PT on body composition and muscle strength in middle school baseball players. The effect sizes (Cohen’s *d*) were calculated by the pooled pre-and post-test standard deviation [35]. Standard interpretations of the effect size were used (Cohen’s *d*: |0.20| ≤ small < |0.50| < medium < |0.80| ≤ large) [36]. Statistical significance was set at 5% for all analyses. SPSS version 25.0 (IBM Japan Ltd., Tokyo, Japan) was used for statistical analysis.

## 3. Results

### 3.1. Change in Body Composition after Pilates Training

Eleven male middle school baseball players aged 14.8 ± 0.42 years underwent anthropometric measurement (Table 2) and body composition indices (Table 3) before and after PT. There were no significant differences in height (*p* = 0.217) and body weight (*p* = 0.205) after PT (Table 2). Comparing the whole-body composition parameters, only the changes in lean mass (*p* < 0.001) and fat-free mass (*p* < 0.001) after PT were significant (Table 3). Lean mass increased from 55.97 ± 5.72 kg to 57.08 ± 5.21 kg in pre-test and post-test values with a medium effect size (*d* = 0.202). The fat-free mass also increased from 59.25 ± 6.25 kg to 60.39 ± 5.56 kg with a small effect size (*d* = 0.192). In contrast, there were no differences in the percentage of fat, fat mass, BMC, and BMD between pre- and post-PT.

**Figure 1:**
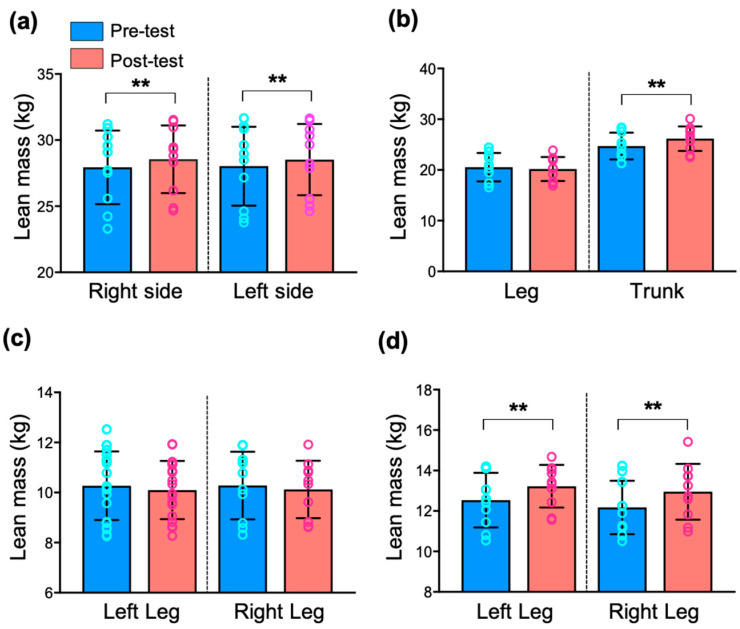
Change in regional lean mass following 8 weeks of Pilates training. (**a**) Lean mass in the right and left sides increased post-test (*p* < 0.01). (**b**) Lean mass in the trunk, but not in the leg, increased post-test (*p* < 0.01). (**c**) There was no significant change in lean mass in the right and left legs between pre- and post-test. (**d**) Trunk lean mass in the right and left sides increased post-test (*p* < 0.01). All values are presented as mean ± SD (n = 11). Note: ** *p* < 0.01, vs. pre-test.

To investigate the contralateral compositional effects of PT in the lower limbs, we further analyzed regional body composition quantification (Figure 1 and Table S1). There was a significant increase in whole-body lean mass on the right and left sides (*p* < 0.001). A significant increase in regional lean mass after PT was observed in the trunk (*p* < 0.001) but not in the leg. In addition, trunk lean mass increased in both the right and left sides (*p* < 0.001).

### 3.2. Changes in Knee Strength after Pilates Training

**Figure 2:**
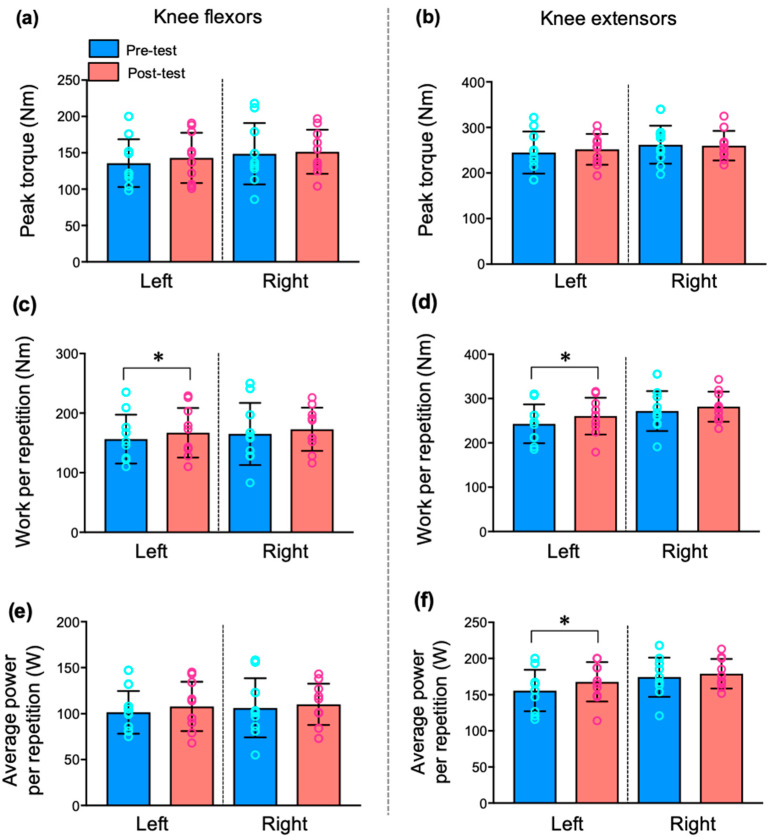
Change in isokinetic knee strength following 8-week Pilates training program. (**a**,**b**) There were no significant changes in peak torque of the knee flexor and extensors. (**c**,**d**) Work per repetition was increased in left knee flexor (*p* < 0.05) and extensors (*p* < 0.05). (**e**,**f**) Average power per repetition was increased in left knee extensors (*p* < 0.05). All values are presented as mean ± SD (n = 11). Note: * *p* < 0.05, vs. pre-test.

To assess the influence of PT on the muscular performance of the knee, we measured the peak torque, work, and average power in the isokinetic test pre- and post- PT (Figure 2 and Table S2). When the pre- and post-test values were compared, there were no significant changes in flexor and extensor peak torque for the right and left knees. However, after PT, the work values of the left knee significantly increased in both the flexor (*p* < 0.05) and extensor (*p* < 0.05). The average power of the knee extensor was significantly increased on the left side (*p* < 0.05) but not on the right side.

## 4. Discussion

Leading sports organizations have released consensus statements about the importance of encouraging adolescents to regularly participate in strength and conditioning programs to promote athletic development and the prevention of injury [37,38]. Therefore, appropriate conditioning programs should be developed using evidence-based information. In this study, we investigated the effectiveness of supervised PT as a conditioning program for adolescent baseball players. The results support our hypothesis that increasing lean mass and muscular strength in the lower limbs and trunk improves body composition after 8 weeks of PT. However, because there was no control group that also underwent PT intervention in this study, we cannot conclude that the training adaptations reported were all a result of the Pilates training.

An accurate assessment of body composition in athletes is important to monitor their sport-specific performance and conditioning. Our previous pilot study showed that PT in-creased muscle mass in adolescent baseball players using a multifrequency bioelectrical impedance (BIA) device [32]. Furthermore, in this study, we precisely evaluated the positive effect of PT on fat and lean mass, not only in the total body but also in specific regional areas, using DEXA [39], which may be a promising tool for assessing athletic performance. DEXA is widely used in athletic populations, and the International Olympic Committee reported that nearly 40% of sports organizations had used DEXA [40,41]. Using DEXA, we found that PT symmetrically increased the lean mass in the trunk. A previous study demonstrated that a rehabilitation program involving injured baseball players increased muscle mass [42], which is similar to the effects of PT described in our study. Our findings may provide some insight into the usefulness of PT in preventing injury by minimizing asymmetries in body movement during sports performance.

**Figure 3:**
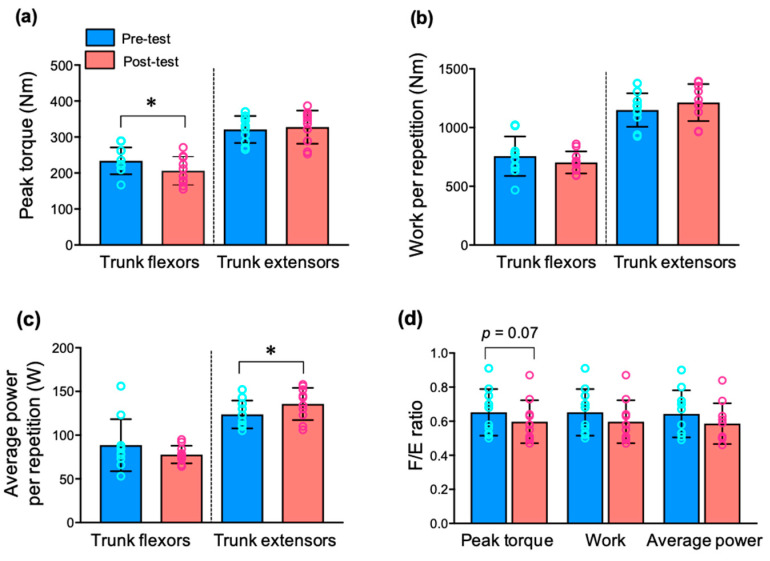
Change in isokinetic trunk strength following 8 weeks of Pilates training. (**a**) There was a significant change in peak torque of trunk flexors (*p* < 0.05). (**b**) There was no significant change in work per repetition of trunk flexors and extensors. (**c**) Average power per repetition in trunk extensors increased in post-test (*p* < 0.05). (**d**) Ratio of trunk flexion/extension strength (F/E) in post-test trended lower than that in pre-test (*p* = 0.07). All values are presented as mean ± SD (n = 11). Note: * *p* < 0.05, vs. pre-test.

Timothy et al. [12] suggested that the development of muscular strength plays an important role in enhancing athletic performance and reducing injury. In baseball, the trunk, as a core part of the human body, plays an important role in the kinetic mechanism of pitching via an efficient transmission of energy and force from the lower limbs to the upper limbs to maximize ball velocity [19]. Moreover, trunk motion is associated with a potential risk of injury to the upper extremities [43,44]. Our study showed an increase in the isokinetic muscular strength of the trunk extensors via intervention with PT (Figure 3c). However, PT decreased the strength of the trunk flexors (Figure 3a). A previous study demonstrated that stretching exercises improved back extensor strength [45]. In adolescent baseball players, this functional movement program, which included body stretching, improved strength and flexibility in motion performance capability [46]. Because of the principal movements of PT, our intervention may be more effective in the trunk extensors. As various training conditions may have positive advantages in improving muscular strength [47], PT may improve performance as well as preventing potential injury.

Kinetic characterization and injury of the upper extremities, including the shoulder and elbow, are a major focus of research in baseball. In terms of efficient energy transfer, the lower extremity is also an important component during the multiple phases of baseball, including throwing, base running, and hitting [48,49]. Therefore, we investigated the effects of PT on knee muscle strength. Notably, the isokinetic strength of the flexor and extensor muscles in the left knee, but not in the right knee, significantly increased after PT (Figure 2), which is consistent with our previous results [32]. This result is interesting because PT was not accompanied by improvements in knee strength bilaterally. Most adolescent players in this study were right-handed pitchers, with the right side being the dominant side. Therefore, the left knee was the lead leg that is defined as the leg contralateral to the throwing arm. According to our results, the strength of the left knee was lower than that of the right knee at pre-test, which is consistent with a significant difference in limb muscle strength between dominant and non-dominant legs [50]. However, PT improved the weak left knee strength to a value similar to that of the right knee. Within the kinetic chain on throwing, the lead leg provides support, absorbing the energy that is eventually transferred during the deceleration and follow-through phases, which are related to injuries [51]. Therefore, PT could be an appropriate training approach to prevent imbalance in both leg extremities.

Trunk strength and stability are critical factors for generating the strong force during throwing or hitting performance in athletic sports. The F/E ratio is a relevant parameter for measuring the functional capacity or asymmetry of the trunk muscle’s strength capacity [52,53]. Usually, the trunk muscle F/E ratio in athletes (0.5–0.7) is lower than that in healthy untrained adults (0.7–0.9), in accordance with increased trunk extensor strength [54,55]. The adolescent athletes in our study showed that the trunk muscle F/E ratio of the peak torque decreased from 0.731 to 0.631 after PT (Figure 3), indicating low trunk flexor muscle strength levels relative to the extensor muscles. To the best of our knowledge, this is the first study to investigate the effect of PT on the F/E ratio of the trunk muscles in adolescent baseball players. The values of the F/E ratio (0.631) after PT are similar to the values assessed in young athletes by Mueller et al. [56]. A previous study reported that higher sport-specific performances showed lower values of F/E ratio in the trunk muscles [52]. Although differences in the trunk strength capacity depend on age and sports specialization in adolescent athlete [57], PT may improve trunk muscle strength and stabilize trunk movements in adolescent baseball players. A significant correlation between trunk strength and bat velocity during the baseball swing has also been reported [58]. Future investigations on the effects of PT on baseball-related performance are warranted.

The present study has several limitations. First, it is difficult to conclude from these results that the positive adaptation effects were all a result of the PT program, because our experiment design did not include a control group without PT intervention. Second, we did not control for lifestyle habits (e.g., food intake, additional exercise, and medical care) of the participants so as not to hinder their healthy growth and development in adolescence. These lifestyle factors may have influenced our results. Lastly, considering sample size, this study had a relatively small sample size, and further studies should include larger sample sizes. Nevertheless, our findings provide preliminary evidence for the potential effects of PT to improve body composition and muscular strength.

## 5. Conclusions

Our results demonstrate that a PT intervention in an off-season conditioning program in adolescent baseball players had the potential to improve lean mass and muscular strength in the knee and trunk. Despite the limitations of our study design mentioned above, our findings contribute up-to-date information for coaches to use in optimizing the physical condition of adolescent baseball players.

## Figures and Tables

**Table 1:** Pilates training program for adolescent athletes.

Stage	Training Period			
	1–2 Weeks	3–4 Weeks	5–6 Weeks	7–8 Weeks
Warm-up	Breathing			
	Hip release			
	Roll-up preparation			
	Arm circles			
Work-out	Single-leg stretches	Roll up	The hundred	The hundred
	Double-leg stretches	Rolling like a ball	Single-leg circles	Side kick: front/back
	Rolling like a ball	Side bend	Leg pulls back and front	Side kick: small circles
	Swimming	Spine stretch forward	Roll over	Rowing
	Obliques	Side leg series	Side bend	Swan
	Spine twist	Criss-cross	Spine stretch forward	Teaser
	Spine stretch forward	Mermaid	Side leg series	Side bend
Cool down	Head nods			
	Hip rolls			
	Breaststroke prep			
	Cat stretch			

**Table 2:** Participant anthropometric measurements.

Variable	Pre	Post	*p*-Value
Age (years)	14.8 ± 0.42		-
Height (cm)	173.8 ± 6.03	174.0 ± 5.88	0.2172
Weight (kg)	79.17 ± 14.11	79.93 ± 12.35	0.2054

Values are the mean ± SD.

**Table 3:** Change in body composition following 8 weeks of Pilates training in adolescent baseball players.

Variable	Pre	Post	*p*-Value	Cohen’s *d* Effect Size
Percentage fat (%)	25.43 ± 8.24	24.98 ± 7.23	0.2737	0.058
Fat mass (kg)	20.22 ± 9.77	19.83 ± 8.20	0.3317	0.043
Lean mass (kg)	55.97 ± 5.72	57.08 ± 5.21 ***	0.0003	0.202
Fat free mass (kg)	59.25 ± 6.25	60.39 ± 5.56 ***	0.0002	0.192
BMC (kg)	3.28 ± 0.40	3.30 ± 0.41	0.1725	0.049
BMD (g/cm^2^)	1.26 ± 0.06	1.25 ± 0.05	0.3200	0.181

Values are the mean ± SD., *** *p* < 0.001 vs. pre-test. Effect size range: |0.20| ≤ small < |0.50| < medium < |0.80| ≤ large. Note: BMC = bone mineral content, BMD = bone mineral density.

## Data Availability

The data presented in this study are available upon request from the corresponding author. The data are not publicly available due to privacy restrictions.

## Supplementary Materials

The following are available online at https://www.mdpi.com/article/10.3390/ijerph191912085/s1, Table S1: Change of in and trunk lean mass following 8-week Pilates training in adolescent baseball players, Table S2: Change in leg and trunk isokinetic strength following 8-week Pilates training in adolescent baseball players.
