# Supplementary material for: Effectiveness of Pilates Training on Body Composition and Isokinetic Muscular Strength in Adolescent Baseball Players
Source: Int J Environ Res Public Health. 2022 Sep 24;19(19):12085. doi: 10.3390/ijerph191912085 (PMC9566218; doi:10.3390/ijerph191912085)
Supplement: Supplementary file 1 [file ijerph-19-12085-s001.zip › ijerph-1898375-supplementary.pdf]

Table S1. Change of leg and trunk lean mass following 8-week of Pilates training in adolescent baseball players

Variable (kg)	Pre	Post	*p*-value	Cohen's *d* effect size
Right leg lean mass	10.28 ± 1.35	10.13 ± 1.47	0.1969	0.106
Left leg lean mass	10.26 ± 1.46	10.07 ± 1.24	0.1425	0.140
Right trunk lean mass	12.18 ± 1.32	12.87 ± 1.33**	0.0005	0.520
Left trunk lean mass	12.54 ± 1.34	13.22 ± 1.05**	0.0003	0.569
Right total lean mass	28.03 ± 2.98	28.52 ± 2.69**	0.0006	0.172
Left total lean mass	27.94 ± 2.78	28.55 ± 2.55**	0.0045	0.228

Values are the mean ± SD., $^{**}p < 0.01$ vs. pre-test. Effect size range: |0.20 | ≤ small < |0.50| < medium < |0.80| ≤ large.

Table S2. Change of leg and trunk isokinetic strength following 8-week of Pilates training in adolescent baseball players

Parameters	Pre	Post	*p*-value	Cohen's *d* effect size
**Right knee flexion**				
Peak torque (Nm)	148.7 ± 42.2	151.4 ± 30.36	0.374	0.074
Work per repetition (Nm)	165.0 ± 52.0	172.9 ± 36.31	0.240	0.178
Average power per repetition (W)	106.3 ± 32.05	110.3 ± 22.52	0.273	0.146
**Left knee flexion**				
Peak torque (Nm)	137.9 ± 31.15	144.2 ± 33.2	0.126	0.195
Work per repetition (Nm)	156.4 ± 40.93	167.1 ± 41.64*	0.027	0.259
Average power per repetition (W)	101.5 ± 23.2	107.9 ± 26.82	0.060	0.255
**Right knee extension**				
Peak torque (Nm)	244.9 ± 46.22	252.0 ± 33.73	0.243	0.177
Work per repetition (Nm)	271.7 ± 45.03	281.8 ± 33.83	0.149	0.256
Average power per repetition (W)	174.2 ± 26.92	178.9 ± 20.28	0.230	0.199
**Left knee extension**				
Peak torque (Nm)	191.7 ± 32.55	199.4 ± 34.17	0.204	0.230
Work per repetition (Nm)	242.9 ± 43.86	260.4 ± 41.5*	0.031	0.409
Average power per repetition (W)	155.7 ± 28.55	167.8 ± 27.00*	0.023	0.435
**Trunk flexion**				
Peak torque (Nm)	233.8 ± 27.35	206.3 ± 39.34*	0.043	0.811
Work per repetition (Nm)	756.3 ± 168.3	702.6 ± 93.75	0.171	0.394
Average power per repetition (W)	88.5 ± 29.82	77.8 ± 10.12	0.122	0.480
**Trunk extension**				
Peak torque (Nm)	321.1 ± 37.58	327.6 ± 46.15	0.326	0.154
Work per repetition (Nm)	1150.0 ± 142.8	1213.0 ± 157.3	0.080	0.419
Average power per repetition (W)	123.7 ± 15.94	135.7 ± 18.42*	0.049	0.696

Values are the mean ± SD., $^{*}p < 0.05$ vs. pre-test. Effect size range: $|0.20| \leq$ small $< |0.50| <$ medium $< |0.80| \leq$ large.
